# Flavanone Glycosides, Triterpenes, Volatile Compounds and Antimicrobial Activity of *Miconia minutiflora* (Bonpl.) DC. (*Melastomataceae*)

**DOI:** 10.3390/molecules27062005

**Published:** 2022-03-21

**Authors:** Nathália Siso Ferreira, Márcia Moraes Cascaes, Lourivaldo da Silva Santos, Mozaniel Santana de Oliveira, Maria das Graças Bichara Zoghbi, Isabella Santos Araújo, Ana Paula Trovatti Uetanabaro, Eloisa Helena de Aguiar Andrade, Giselle Maria Skelding Pinheiro Guilhon

**Affiliations:** 1 Programa de Pós-Graduação em Química, Universidade Federal do Pará, Avenida Augusto Corrêa 01, Belém 66075-110, PA, Brazil; nathisisof@gmail.com (N.S.F.); lss@ufpa.br (L.d.S.S.); eloisa@museu-goeldi.br (E.H.d.A.A.); giselle@ufpa.br (G.M.S.P.G.); 2 Laboratório Adolpho Ducke, Coordenação de Botânica, Museu Paraense Emílio Goeldi, Avenida Perimetral, 1901, Belém 66077-830, PA, Brazil; mozaniel.oliveira@yahoo.com.br (M.S.d.O.); zoghbi@museu-goeldi.br (M.d.G.B.Z.); 3 Programa de Pós-Graduação em Biotecnologia, Universidade Estadual de Feira de Santana, Avenida Transnordestina s/n, Feira de Santana 44036-900, BA, Brazil; araujo_isabella@yahoo.com.br (I.S.A.); uetanabaro@yahoo.com (A.P.T.U.)

**Keywords:** pinocembroside, pinocembroside derivative, ursolic acid, essential oils, antimicrobial assays

## Abstract

Chemical composition of the essential oils and extracts and the antimicrobial activity of *Miconia minutiflora* were investigated. The flavanone glycosides, pinocembroside and pinocembrin-7-O-[4″,6″-HHDP]-β-D-glucose, were identified, along with other compounds that belong mainly to the triterpene class, besides the phenolics, gallic acid and methyl gallate. Sesquiterpenes and monoterpenes were the major compounds identified from the essential oils. Screening for antimicrobial activity from the methanolic extract of the leaves showed that the MIC and MMC values against the tested microorganisms ranged from 0.625 to 5 mg·mL^−1^ and that the extract was active against microorganisms, *Staphyloccocus* *aureus*, *Escherichia coli,* and *Bacillus* *cereus*.

## 1. Introduction

Melastomataceae comprises 166 genera and around 4500 species [1]. About 1470 species were cataloged in Latin America distributed in countries such as Brazil, Uruguay, Mexico, Argentina, Colombia, Ecuador, and Venezuela [2]. More than 1400 species within 69 genera occur in Brazil spread throughout the Amazon to the Uruguay frontier [3]. *Miconia* is the most representative genus of Melastomataceae, with a wide distribution in the American continent including 1057 species and representing the largest genus of woody flowering plants with a distribution restricted to tropical America [4].

Some *Miconia* species were widely used in folk medicine to treat diarrhea and stomachache [5]. Isolated compounds and *Miconia* extracts have demonstrated diverse pharmacological activities. The aqueous extract of *M. latecrenata* (DC.) Naudin leaves showed a high antioxidant effect and high antibacterial activity [4]. The cytotoxic and mutagenic potential of extracts from *M. cabucu* Hoehne, *M. rubiginosa* (Bonpl.), *M. stenostachya* DC., and *M. albicans* (Sw.) Steud were investigated and the results confirmed the safe use of *Miconia* extracts and reinforced the therapeutic properties and their protective effects on doxorubicin-induced mutagenicity [6]. The ethanolic leaf extract of *M. albicans* shows an anti-arthritic profile [7]. The ethanolic extract of *M. willdenowii* Klotzsch ex Naudin showed schistosomicidal [8], antimicrobial activities, and anti-*L. amazonensis* effects, as well as evidence that the most abundant constituent, the benzoquinone derivative primin, is the major bioactive metabolite [9].

Previous studies on *Miconia* species have revealed the presence of triterpenes [10], tannins [4], flavanone glycosides [11], benzoquinones [8], saponins, and leucoanthocyanins [12].

This work reports the chemical composition of extracts and essential oils and the antimicrobial activity of the methanolic extract from the leaves of *Miconia minutiflora* (Bonpl.) DC. A survey of the literature shows one study on the volatiles of *M. minutiflora* inflorescences, which were characterized by the presence of α-copaene and β-caryophyllene as major constituents [13]. Another study on *M. minutiflora* showed the anti-inflammatory and antinociceptive effects of the leaf methanol extract; in the same work, the authors tentatively identified the compounds casuarinin (4 isomers), ellagic acid, HHDP-galloylglucose (one isomer), myricetin-galloyl-deoxihexoside (one isomer), myruianthic acid (two isomers), and arjunolic acid (seven isomers) using UPLC-DAD-QTOF-MS/MS [14]. Effects of the extracts of seven *Miconia* species, including *M. minutiflora*, were evaluated on *Lactuca sativa* seeds and seedlings growth, the extract of *M. minutiflora* showed no allelopathic effects on the rootlets of the tested plant [15].

## 2. Results

### 2.1. Non-Volatiles

Chemical investigation of *M. minutiflora* leaves’ extract led to a mixture containing, mainly, a mixture of hydrocarbons (octacosane 16.95%, nonacosane 3.18%, triacontano 39.66%, untriacontano 5.13%, and dotriacontano 33.15%) (M1), the triterpene squalene [16] (S1), a mixture of the terpenoids β-amirin, α-amyrin, taraxerol and lupeol [17] (M2), a mixture of phytol [18], β and α-amyrin [19], and fatty acids (palmitic and linoleic acids) [20] (M3), and a mixture of the steroids, stigmasterol [21], spisnasterol [22] and sitosterol [23] (M4). Fractionation of the methanol extract from the leaves led to a mixture of the phenolics compounds, gallic acid and methyl gallate [24] (M5), and isolation of the flavanones, pinocembroside [25] (S4) and pinocembrin-7-O-[4″,6″-HHDP]-β-glucose [26] (S5).

**Figure 1:**
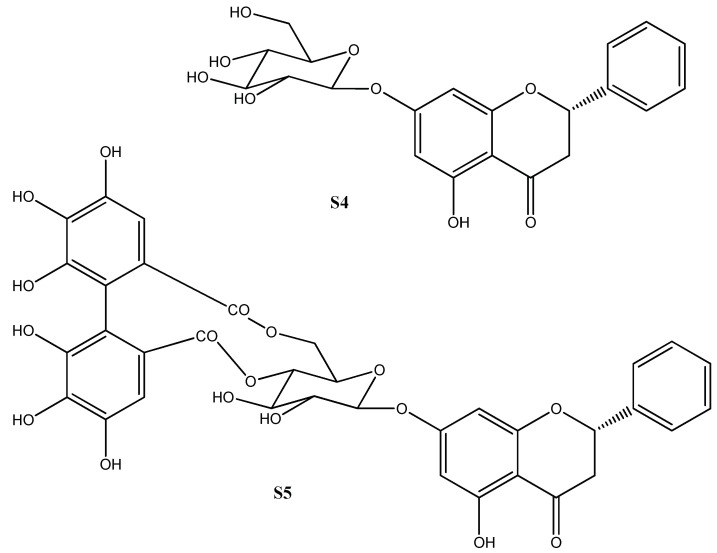
Chemical structure of isolated compounds, **S4** and **S5**.

Study of the *M. minutiflora* stems led to the isolation of sistoterol (S2), a mixture of triterpenes β and α-amyrin and fatty acids (M6), besides that a mixture of fatty acids (M7), besides a mixture of the steroids, stigmasterol and sitosterol (M8), and the triterpene ursolic acid [27] (S3). The structures of the major isolated compounds from the leaves’ extracts, pinocembroside (S4) and pinocembrin-7-O-[4″,6″-HHDP]-β-glucose (S5), are shown in Figure 1. Although the chemical composition of *M. minutiflora* found in this research is in accordance with other *Miconia* species, this is the first time that flavanone glycosides (pinocembroside and pinocembrin-7-O-[4″,6″-HHDP]-β-glucose) were isolated from *Miconia*.

### 2.2. Volatile Compounds

The percentage of the compounds identified in the essential oils and the sequence of their retention indices are listed in Table 1. Leaves, primary and secondary branches of *M. minutiflora* yield below 0.1% of essential oils, just enough to perform the chemical characterization. In total, 67 compounds were identified. The major constituents in the essential oils from the leaves were (3*Z*)-hexenol (9.73%), 1-octen-3-ol (8.16%), (3*Z*)-hexenylbutanoate (9.77%), cis-3-hexenyl isovalerate (8.65%), (3*Z*)-hexenyl hexanoate (10.91%), and phytol (7.34%). (9*Z*,12*Z*)-Octadecadienoic acid (48.71%) and *n*-hexadecanoic acid (32.65%) were the major compounds in the primary branches and in the secondary branches, the main compounds were *n*-hexadecanoic acid (42.75%), (9*Z*,12*Z*)-octadecadienoic acid (35.65%), and dodecanoic acid (6.36%).

Zoghbi and coworkers (2000) [13] obtained the volatile concentrate by micro-simultaneous distillation extraction from *M. minutiflora* inflorescences using pentane as a solvent and the main compounds identified were the sesquiterpenes α-copaene (22.82%) and β-caryophyllene (14.46%). The volatiles of the inflorescences of two other species of *Miconia*, also extracted by micro-simultaneous distillation extraction, shown as major compounds were (*E*,*E*)-α-farnesene (14.7%) and *p*-cymene (10.3%) in *M. ciliata* (Rich.) DC., while α-copaene (32.9%) and nonanal (18.5%) were the major constituents of *M. rubiginosa* (Bonpl.) DC. [13]. The main compounds identified in the essential oil of the aerial parts of *M. ferruginata* were the sesquiterpenes, β-caryophyllene (56.2%) and α-humulene (7.3%), the hydrocarbon, 8-heptadecene (16.8%), and the alcohol, l 1-octen-3-ol (9.5%) [28].

### 2.3. Antimicrobial Activity

The minimal inhibitory concentration (MIC) and minimum microbicidal concentration (MMC) of the methanol extract from *M. minutiflora* against the tested microorganisms ranged from 0.625 to 5 mg·mL^−1^, these data are shown in Table 2. The methanol extract was active against chloramphenicol resistant microorganisms, E. coli CCMB 261 (MIC = 0.625 and MMC = 1.25 mg·mL^−1^), *S. aureus* CCMB 262 (MIC = 0.625 and MMC = 1.25 mg·mL^−1^), *S. aureus* CCMB 263 (MIC = 0.625 and MMC = 1.25 mg·mL^−1^), *S. aureus* CCMB 285 (MIC = 0.625 and MMC = 1.25 mg·mL^−1^), *B. cereus* CCMB 282 (MIC = 0.625 and MMC = 1.25 mg·mL^−1^), and a nystatin resistant *C. parapsilosis* CCMB 288 (MIC = 5 and MMC = 10 mg·mL^−1^).

Rodrigues and coworkers (2008) tested the dichloromethane extract of *M. cabucu* against *C. albicans* and obtained an MIC value of 1.5 mg·mL^−1^ and the methanol extract of *M. stenostachya* against *B. cereus* showed an MIC of 3.0 mg·mL^−1^ [29]. The ethanol extract of *M. albicans* and *M. rubiginosa* showed antimicrobial activity using the well diffusion method [30].

Some compounds obtained from *Miconia* species also demonstrated antimicrobial activity such as the mixture of ursolic and oleanolic acids isolated from *M. ferruginata* leaves that was active against *S. aureus*, *E. coli*, *Bacillus subtilis*, and *Pseudomonas aeruginosa* [31]. The antifungal activity of the isolated compound pinocembroside (S4) was previously described using in vitro mycelial growth of Penicillium italicum, showing an MIC and minimum fungicidal concentration (MFC) of 200 and 800 mg·L^−1^, respectively, [32] and against Penicillium digitatum, with a half-maximal effective concentration (EC_50_), MIC, and MFC of 120.3, 200, and 400 mg·L^−1^, respectively [33]. Gallic acid [34] and methyl gallate also have shown antimicrobial activity [35], gallic acid has already been obtained from extracts from *M. rubiginosa* [6], while methyl gallate was obtained from the ethanolic extract from Monochaetum multiflorum (Bonpl.) (Melastomataceae) [36]. In addition, pinocembrin-7-O-[4″,6″-HHDP]-β-glucose showed antibacterial activity on *E. coli*, *S. aureus*, *Enterococcus faecalis*, *Lactobacillus rhamnosus*, and *Bacillus subtilis* [37].

The antimicrobial activity observed for the methanol extract of *M. minutiflora* can be explained by the presence of substances in the studied extracts indicating that this species is a valuable source for the discovery of new antimicrobial products.

## 3. Materials and Methods

### 3.1. Plant Material

Leaves and stems of *M. minutiflora* were collected in the Municipality of Belém (Estrada do Paiol, Km 5), State of Pará, Brazil in June 2010 for the study of its non-volatile compounds. A voucher specimen was identified and deposited at the Herbarium of the Museu Paraense Emílio Goeldi (Belém—Pará—Brazil) under the reference number MG-204.906. Another collection (leaves, primary and secondary branches) was taken in the same municipality at the Museu Paraense Emílio Goeldi—research campus in November 2014 for the identification of the volatile compounds; this specimen was identified at the same herbarium by comparison with the same voucher.

### 3.2. Extraction, Isolation, and Identification of the Non-Volatile Compounds

Leaves (2.00 Kg) and stems (2.00 Kg) of *M. minutiflora* were extracted by maceration with hexane (7 days × 2) and MeOH (14 days × 2) at room temperature. The filtrates were concentrated under reduced pressure to yield the hexane extracts (30.00 g of leaves extract and 7.00 g of stems extract) and the methanolic extracts (267.00 g of leaves extract and 118.00 g of stems extract). Part of the methanolic extracts (40.00 g each) was suspended in MeOH-H_2_O 3:1 and extracted with CH_2_Cl_2_, EtOAc, and *n*-BuOH yielding the leaves phases (CH_2_Cl_2_ phase: 7.00 g, EtOAc phase: 13.87 g, *n*-BuOH: 8.00 g) and the stems phases (CH_2_Cl_2_ phase: 3.00 g, EtOAc phase: 2.54 g, *n*-BuOH: 1.19 g) of the methanolic extracts.

The hexane extracts of the leaves (20.00 g) and of the stems (6.00 g), the CH_2_Cl_2_ phase of the stems (3.00 g), and the EtOAc phase of the leaves (14.03 g) were purified using column chromatography (CC) over silica gel using mixtures of hexane–EtOAc and EtOAc–MeOH with increasing polarity. When necessary, the resulting fractions were rechromatographed using similar techniques. The hexane extract of the leaves afforded M1 (1401 mg), S1 (772 mg), M2 (20 mg), M3 (21 mg), and M4 (56 mg). The hexane extract of the stems afforded M6 (44 mg), M7 (17 mg), and S2 (205 mg). The CH_2_Cl_2_ phase from stems afforded M8 (38 mg) and S3 (7 mg). Fractions of the EOAc phase of the leaves eluted with EtOAc–MeOH 50% and MeOH were reunited and fractionated by CC affording MM1 (100 mg) and MM2 (600 mg). Part of fraction MM1 (10 mg) was purified on an SPE cartridge eluted with 3 × 1 mL ACN:H_2_O 90:10 yielding M5 (4 mg). Fraction MM2 was submitted to fractioning by semipreparative HPLC using as eluent the system ACN:H_2_O 38:62 and flow of 4.7 mL.min^−1^ yielding S4 (18 mg) and S5 (19 mg). Spectra and spectral data (^1^H, ^13^C) of S4 and S5 are provided in the Supplementary Material along with chemical and instruments. 

### 3.3. Extraction of the Essential Oils

Samples of leaves, primary and secondary branches (120 g) were hydrodistilled for 3 h, using a Clevenger-type apparatus with maintenance of the refrigeration water at 15 °C in accordance with the works described in the literature [38,39].

#### Analysis of the Essential Oils

The chemical composition of the volatile compounds of the *Miconia minutiflora* (Bonpl.) DC. (*Melastomataceae*) was analyzed using gas chromatography coupled to mass spectrometry, using a Thermo DSQ-II system equipped with a DB-5MS silica capillary column (30 m × 0.25 mm; 0.25 mm). For this analysis, the same protocols described previously by our research group were followed [40,41]. The volatile compounds present in the essential oil were identified by comparison with the literature [42,43].

### 3.4. Antimicrobial Analysis

The analysis of the antimicrobial potential of the methanolic extract from the leaves of *M. minutiflora* was realized against the microorganism *C. parapsilosis* CCMB 288 (resistant to anfoterycin-B), *C. albicans* CCMB 286, *C. albicans* CCMB 266, *B. cereus* CCMB 282, *P. aeruginosa* CCMB 268, *Salmonella* sp. CCMB 281, *S. aureus* CCMB 263, *S. aureus* CCMB 285, *S. aureus* CCMB 262 (resistant to streptomycin and dihydrostreptomycin), and *E. coli* CCMB 261 (sensitive to trimetoprime and resistant to sulphonamide).

#### 3.4.1. Well Diffusion Test

The antimicrobial activity of the methanol extract was first evaluated using the well diffusion test as follows. A swab of the microorganism was transferred to 6 mL of a 0.45% saline solution and the resulting suspension was adjusted to 0.1 mL of a 1.5 × 108 cels·mL^−1^ (bacteria) and 1.5 × 105 cels·mL^−1^ (yeast). The cells suspension was added to 120 mL of MHA. The resulting mixture was transferred to Petri dishes (100 mm). After cooling the mixture, six equidistant wells (6 mm in diameter) received 65 μL of the methanol extract, at 200 mg·mL^−1^ in DMSO-water 1:1. Positive controls were chloramphenicol at 30 μg·mL^−1^ for bacteria and nystatin at 10 μg·mL^−1^ for yeast. The negative control was DMSO. Petri dishes were incubated at 37 °C for 24 h (bacteria) and at 28 °C for 48 h (yeast). The results were reported as the diameter of the zone of inhibition (in mm) (CLSI, 2003, with adaptations) [44].

#### 3.4.2. Minimum Inhibitory Concentration (MIC) and Minimal Microbicidal Concentration (MMC)

The minimum inhibitory concentration (MIC) and minimal microbicidal concentration (MMC) of the methanol extract of the leaves of *M. minutiflora* were performed following the same protocols described in previous works [45,46].

## 4. Conclusions

This study showed that the extracts of *M. minutiflora* are an important source of glycosylated flavanones, which are very often identified from *Miconia*. This is the first time that the flavanones, pinocembroside and pinocembrin-7-O-[4″,6″-HHDP]-β-glucose, were isolated from a *Miconia* species. Compounds (3*Z*)-hexenol, 1-octen-3-ol, (3*Z*)-hexenylbutanoate, cis-3-hexenyl isovalerate, (3*Z*)-hexenyl hexanoate, and phytol were the major constituents of the essential oils from the leaves, while *n*-hexadecanoic acid and (9*Z*,12*Z*)-octadecadienoic acid were the major constituents from the primary branches and (9*Z*,12*Z*)-octadecadienoic acid, dodecanoic acid, tetradecanoic acid and *n*-hexadecanoic acid were the major constituents from the secondary branches. The antimicrobial screening showed that the leaves’ methanol extract is active against *E. coli*, *S. aureus,* and *B. cereus* and these activities can be in part explained by the presence of known bioactive compounds in the extracts.

## Figures and Tables

**Table 1:** Constituents (%) identified in the essential oils of the leaf, primary and secondary branches of *Miconia minutiflora*.

RI_L_	RI_C_	Constituents	Leaf	Branch-1	Branch-2
801	799	Hexanal	0.55	0.03	0.14
846	846	(2*E*)-Hexenal	0.38	0.04	
850	851	(3*Z*)-Hexenol	9.73	0.03	0.1
854	857	(2*E*)-Hexenol	0.77		
863	864	Hexanol	1.39		0.1
907	908	(2*E*,4*E*)-Hexadienal	0.52		
947	914	(2*E*)-Heptenal	0.15		
952	953	Benzaldehyde	0.33	0.02	0.05
959	960	Heptanol	0.25		
974	974	1-Octen-3-ol	8.16	0.15	
981	981	6-methyl-5-Hepten-2-one	0.27		
984	985	3-*p*-Menthene	0.29		
989	990	6-methyl-5-Hepten-2-ol	0.28		0.05
1005	1005	(2*E*,4*E*)-Heptadienal	0.32		
1004	1008	(3*Z*)-Hexenylacetate	0.54		
1024	1026	Limonene	0.25	0.05	0.07
1036	1038	Benzeneacetaldehyde	0.07		0.09
1044	1044	(*E*)-β-Ocimene	0.35		
1063	1063	*n*-Octanol	0.23		
1086	1086	Terpinolene	0.14		
1089	1090	*p*-Cymenene	0.28		
1095	1096	Linalool	1.19		
1100	1100	Nonanal	0.25	0.1	0.15
1102	1115	(2*E*,4*E*)-Octadienal	0.07		
1142	1142	(3*Z*)-Hexenylisobutanoate	0.75		
1152	1152	(3*Z*)-Nonen-1-ol	0.44		
1157	1157	(2*E*)-Nonen-1-al	0.15	0.11	0.12
1184	1183	(3*Z*)-Hexenylbutanoate	9.77		
1191	1186	Hexylbutanoate	0.37	0.05	
1186	1188	α-Terpineol	0.72	0.13	
1196	1196	Safranal	0.17		
1201	1202	Decanal			0.13
1210	1210	(2*E*,4*E*)-Nonadienal			0.05
1232	1226	*cis*-3-Hexenyl isovalerate	8.65		
1249	1248	Geraniol	0.13		
1260	1260	(2*E*)-Decenal	0.25		0.05
1279	1275	*cis*-3-hexenyl valerate	0.26		
1285	1286	Safrole	0.18		0.09
1293	1294	2-Undecanone	0.28		
1315	1315	(2*E*,4*E*)-Decadienal	0.27	0.08	
1330	1330	Hexyl tiglate	0.26		
1364	1365	Decanoic acid			0.11
1374	1374	α-Copaene	0.97		
1383	1382	(*E*)-β-Damascenone	0.57		
1378	1385	(3*Z*)-Hexenyl hexanoate	10.91		
1417	1417	(*E*)-Caryophyllene	2.66		
1428	1428	(*E*)-α-Ionone	0.78		
1432	1432	*trans*-α-Bergamotene	0.19		
1453	1453	Geranyl acetone	0.72		0.05
1452	1454	α-Humulene	0.98		
1487	1488	(*E*)-β-Ionone	1.89		0.05
1495	1495	2-Tridecanone	0.34		
1505	1505	(*E*,*E*)-α-Farnesene	0.36		
1514	1515	β-Curcumene	1.28		
1561	1562	*E*-Nerolidol	0.69		
1565	1566	Dodecanoic acid	0.91	2.3	6.36
1565	1568	(3*Z*)-Hexenyl benzoate	1.93		
1594	1595	Ethyl dodecanoate	0.31		
1722	1725	Tetradecanoic acid	0.59	2.6	4.48
1946	1940	Isophytol	0.34	0.29	
1942	1941	Phytol	7.34		1.41
1959	1959	*n*-Hexadecanoic acid	1.83	32.65	42.75
2029	2117	(9*Z,*12*Z*)-Octadecadienoic acid		48.71	35.65
2124	2130	Octadecanoic acid		0.1	1.48
2500	2501	Pentacosane	0.2		0.44
2600	2603	Hexacosane	0.12	0.72	0.2
2700	2705	Heptacosane	0.12	0.38	0.09
Total			85.44	88.54	94.26

RI_C_ = Calculated retention index; RI_L_ = Literature retention index; Branch-1: primary branches; Branch-2: secondary branches.

**Table 2:** Antimicrobial potential of the methanol extract of *Miconia minutiflora* leaves.

Microorganism	MIC (mg·mL^−1^)	MMC (mg·mL^−1^)	Control Nist/Chlorf DMSO (mg·mL^−1^)	
*Escherichia coli* CCMB 261	0.625	1.25	R	5.00
*Pseudomonas aeruginosa* CCMB 268	1.25	2.5	0.31	5.00
*Salmonella* sp. CCMB 281	1.25	2.5	0.16	5.00
*Staphylococcus aureus* CCMB 262	0.625	1.25	0.31	5.00
*S. aureus* CCMB 263	0.625	1.25	0.31	10.00
*S. aureus* CCMB 285	0.625	1.25	R	10.00
*Bacillus cereus* CCMB 282	0.625	1.25	0.16	5.00
*Candida albicans* CCMB 286	2.5	5	0.63	10.00
*C. albicans* CCMB 266	2.5	5	0.08	10.00
*C. parapsilosis* CCMB 288	5	10	R	10.00

R: resistant, Nyst: nystatin, Chlorf: chloramphenicol.

## Data Availability

Not applicable.

## Supplementary Materials

The following supporting information can be downloaded at: https://www.mdpi.com/article/10.3390/molecules27062005/s1. Chemical and instruments, spectra and spectral data (^1^H, ^13^C) of pinocembroside (**S4**) and pinocembrin-7-O-[4″,6″-HHDP]-β-glucose (**S5**), 300 MHz, DMSO-d_6_.
