# Supplementary material for: Flavanone Glycosides, Triterpenes, Volatile Compounds and Antimicrobial Activity of Miconia minutiflora (Bonpl.) DC. (Melastomataceae)
Source: Molecules. 2022 Mar 21;27(6):2005. doi: 10.3390/molecules27062005 (PMC8954877; doi:10.3390/molecules27062005)
Supplement: Supplementary file 1 [file molecules-27-02005-s001.zip › molecules-1623088-supplementary.pdf]

Supplementary Material

Flavanone Glycosides, Triterpenes, Volatile Compounds and Antimicrobial Activity of *Miconia minutiflora* (Bonpl.) DC. (*Melastomataceae*)

Nathália Siso Ferreira[1], Márcia Moraes Cascaes[1*], Lourivaldo Silva Santos[1], Mozaniel Santana de Oliveira[2], Maria das Graças Bechara Zoghbi[2], Isabella Santos Araújo[3], Ana Paula T. Uetanabaro[3], Eloisa Helena de Aguiar Andrade[1,2] and Giselle Maria Skelding Pinheiro Guilhon[1]

[1] Programa de Pós-graduação em Química, Universidade Federal do Pará, Avenida Augusto Corrêa 01, Belém, Pará, Brazil, 66075-110; nathisisof@gmail.com (N.S.F); cascaesmm@gmail.com (M.M.C); eloisa@museu-goeldi.br (E.H.A.A); lss@ufpa.br (L.S.S.); giselle@ufpa.br (G.M.S.P.G.);
[2] Laboratório Adolpho Ducke, Coordenação de Botânica, Museu Paraense Emílio Goeldi, Avenida Perimetral, 1901, 66077-830, Belém, Pará, Brazil; zoghbi@museu-goeldi.br (M.G.B.Z.); mozaniel.oliveira@yahoo.com.br (M.S.O.)
[3] Universidade Estadual de Santa Cruz, Rodovia Jorge Amado Km 16 Ilhéus, Bahia, Brazil, 45662-900; uetanabaro@yahoo.com (A.P.T.U.);
[4] Programa de Pós-graduação em Biotecnologia, Universidade Estadual de Feira de Santana, Avenida Transnordestina s/n Feira de Santana, Bahia, Brazil, 44036-900; araujo_isabella@yahoo.com.br (I.S.A.)
* Correspondence: cascaesmm@gmail.com; Tel.: Tel.: +55-91-3201-8099

Abstract: Chemical composition of the essential oils and extracts and the antimicrobial activity of *Miconia minutiflora* were investigated. The flavanone glycosides pinocembroside and pinocembrin-7-O-[4'',6''-HHDP]-β-D-glucose were identified, along with other compounds belong mainly to triterpene class, beside the phenolic gallic acid and methyl gallate. Sesquiterpenes and monoterpenes were the major compounds identified from the essential oils. Screening for antimicrobial activity from the methanolic extract leaves showed the MIC and MMC values against the tested microorganisms ranged from 0.625 to 5 $mg.mL^{-1}$ and was active against microorganisms *Staphyloccocus aureus*, *Escherichia coli* and *Bacillus cereus*.

Keywords: Pinocembroside; Pinocembroside derivative; Ursolic acid; Essential oils; Antimicrobial assays

---

Chemical and Instruments

Methanol (MeOH), EtOAc, CH2Cl2, *n*-BuOH, acetonitrile (ACN) and hexane, HPLC, ACS or PA grade, were purchased from Tedia, Synth, Quimex or Isofar. Classic column chromatographic (CC) separations were performed on silica gel 70-230 mesh from Vetec or Merck using mixtures of hexane, EtOAc and MeOH as eluentes. Fractions were monitored by thin layer chromatography (TLC) on silica gel GF254 from Merck or TLC silica gel from Vetec. NMR spectra were acquired in $CDCl_3$, CD_3OD, C_5D_5N or DMSO-d_6 from Tedia, on a Mercury 300 instrument with chemical shift referencing performed with internal solvent resonances calibrated to TMS. Semipreparative HPLC was carried on a Varian liquid chromatograph with UV detector, ProStar 335, using a Phenomenex Gemini C18 column (250 mm × 10 mm, 5 μm).

Spectral data

Squalene ^{1}H NMR ($CDCl_3$): δ_H 1.68 and 1.60 (3H, *s*, H-1, H-24, H-25, H-26, H-27, H-28, H-29, H-30), 5.12 (1H, *m*, H-3, H-7, H-11, H-14, H-18, H-22) and 2.06 (H-4, H-5, H-8, H-9, H-12, H-13, H-16, H-17, H-20, H-21); ^{13}C NMR ($CDCl_3$): δ_C 25.7 (C-1), 131.2 (C-2), 124.4 (C-3), 26.8 (C-4), 39.7 (C-5), 135.1 (C-6), 124.3 (C-7), 26.6 (C-8), 39.7 (C-9), 134.9 (C-10), 124.3

(C-11), 28.3 (C-12), 28.3 (C-13), 124.3 (C-14), 134.9 (C-15), 39.7 (C-16), 26.6 (C-17), 124.3 (C-18), 135.1 (C-19), 39.7 (C-20), 26.8 (C-21), 124.4 (C-22), 131.2 (C-23), 25.7 (C- 24), 17.7 (C-25), 16.0 (C-26), 16.0 (C-27), 16.0 (C-28), 16.0 (C-29), 17.7 (C-30).

β-Amyrin ^{1}H NMR ($CDCl_3$): δ_H 5.18 (1H, *t*, *J*=3.6 Hz, H-12), 3.19 (1H, *m*, H-3); ^{13}C NMR ($CDCl_3$): δ_C 38.6 (C-1), 27.2 (C-2), 79.0 (C-3), 38.8 (C-4), 55.1 (C-5), 18.4 (C-6), 32.6 (C-7), 38.8 (C-8), 47.6 (C-9), 36.9 (C-10), 23.5 (C-11), 121.7 (C-12), 145.2 (C-13), 41.7 (C-14), 26.1 (C-15), 26.9 (C-16), 32.5 (C-17), 47.2 (C-18), 46.8 (C-19), 31.1 (C-20), 34.7 (C-21), 37.1 (C-22), 28.1 (C-23), 15.5 (C- 24), 15.6 (C-25), 16.8 (C-26), 26.0 (C-27), 28.4 (C-28), 33.3 (C-29), 23.7 (C-30).

α-Amyrin ^{1}H NMR ($CDCl_3$): δ_H 5.12 (1H, *t*, *J*=3.5 Hz, H-12), 3.19 (1H, *m*, H-3); ^{13}C NMR ($CDCl_3$): δ_C 38.6 (C-1), 27.2 (C-2), 79.0 (C-3), 38.5 (C-4), 55.1 (C-5), 18.4 (C-6), 32.9 (C-7), 40.0 (C-8), 47.7 (C-9), 36.9 (C-10), 23.3 (C-11), 124.4 (C-12), 139.3 (C-13), 42.0 (C-14), 28.7 (C-15), 26.6 (C-16), 33.7 (C-17), 59.0 (C-18), 39.8 (C-19), 39.7(C-20), 31.2 (C-21), 41.5 (C-22), 28.0 (C-23), 15.6 (C- 24), 15.6 (C-25), 16.8 (C-26), 23.3 (C-27), 28.1 (C-28), 17.5 (C-29), 21.4 (C-30).

Taraxerol ^{1}H NMR ($CDCl_3$): δ_H 5.53 (1H, *dd*, *J*=8.0 Hz, H-15), 3.19 (1H, *m*, H-3).

Lupeol ^{1}H NMR ($CDCl_3$): δ_H 4.68 (1H, *s*, H-29a), 4.56 (1H, *s*, H-29b) 3.19 (1H, *m*, H-3).

Phytol ^{1}H NMR ($CDCl_3$) : δ_H 4.2 (d, *J*=7.0 Hz), 1.60.

Palmitic acid ^{1}H NMR ($CDCl_3$): δ_H 1.25 (2H, *s*), 2.34 (2H, t, *J*=7.4 Hz).

Linoleic acid ^{1}H NMR ($CDCl_3$): δ_H 1.25 (2H, *s*), 2.34 (2H, *t*, *J*=7.4 Hz), 5.34 (1H, *m*)

Sitosterol ^{1}H NMR ($CDCl_3$): δ_H 3,51 (1H, *m*, H-3), 5,33 (1H, *d*, *J*=5,4 Hz), 4,96-5,18 (1H, *m*); ^{13}C NMR ($CDCl_3$): δ_C 37.2 (C-1), 31.6 (C-2), 71.7 (C-3), 42.2 (C-4), 140.7 (C-5), 121.7 (C-6), 31.9 (C-7), 31.9 (C-8), 50.1 (C-9), 36.6 (C-10), 21.1 (C-11), 39.8 (C-12), 42.2 (C-13), 56.7 (C-14), 24.3 (C-15), 28.2 (C-16), 56.7 (C-17), 11.8 (C-18), 19.4 (C-19), 36.1 (C-20), 18.8 (C-21), 34.0 (C-22), 26.0 (C-23), 45.9 (C- 24), 29.0 (C-25), 19.8 (C-26), 19.0 (C-27), 23.0 (C-28), 12.0 (C-29).

Spisnasterol ^{1}H NMR ($CDCl_3$): δ_H 3,51 (1H, *m*, H-3), 5,33 (1H, *d*, *J*=5,4 Hz), 4,96-5,18 (1H, *m*); ^{13}C NMR ($CDCl_3$): δ_C 37.2 (C-1), 31.6 (C-2), 71.7 (C-3), 40.1 (C-4), 29.0 (C-5), 38.0 (C-6), 117.0 (C-7), 138.2 (C-8), 49.0 (C-9), 34.0 (C-10), 21.1 (C-11), 39.8 (C-12), 42.2 (C-13), 55.8 (C-14), 23.0 (C-15), 28.2 (C-16), 55.9 (C-17), 12.0 (C-18), 13.0 (C-19), 40.1 (C-20), 21.0 (C-21), 138.2 (C-22), 129.2 (C-23), 51.8 (C- 24), 31.9 (C-25), 19.0 (C-26), 21.1 (C-27), 25.6 (C-28), 12.0 (C-29).

Stigmasterol ^{1}H NMR ($CDCl_3$): δ_H 3,51 (1H, *m*, H-3), 5,33 (1H, *d*, *J*=5,4 Hz), 4,96-5,18 (1H, *m*); ^{13}C NMR ($CDCl_3$): δ_C 37.2 (C-1), 31.8 (C-2), 71.7 (C-3), 140.7(C-4), 121.7 (C-5), 42.2 (C-6), 31.9 (C-7), 32.0 (C-8), 50.1 (C-9), 36.6 (C-10), 21.1 (C-11), 39.8 (C-12), 42.2 (C-13), 56.7 (C-14), 24.3 (C-15), 28.2 (C-16), 56.7 (C-17), 12.0 (C-18), 19.4 (C-19), 40.1 (C-20), 21.0 (C-21), 138.2 (C-22), 129.2 (C-23), 51.8 (C- 24), 31.9 (C-25), 21.1 (C-26), 19.3 (C-27), 25.6 (C-28), 12.0 (C-29).

Gallic acid ^{1}H NMR (CD_3OD): δ_H 7.04 (1H, *s*, H-3 and H-6),

Methyl gallate ^{1}H NMR (CD_3OD): δ_H 7.04 (1H, *s*, H-3 and H-6), 3.80 (1H, *s*, CH3)

Pinocembroside (**S-4**) ^{1}H NMR (DMSO-d6): Flavonoid unit δ_H 5.65 (1H, *dd*, *J*=12.8 and 3.1 Hz, H-2), 2.85 (1H, *dd*, *J*=17.3 and 3.1 Hz, H-3a), 3.32 (1H, *dd*, *J*=17.3 and 12.8 Hz, H-3b), 6.20 (1H, *d*, *J*=2.1 Hz, H-6), 6.15 (1H, *d*, *J*=2.1 Hz, H-8), 7.53 (1H, *m*, H-2′), 7.43 (1H, *m*, H-3′), 7.43 (1H, *m*, H-4′), 7.43 (1H, *m*, H-5′), 7.53 (1H, *m*, H-6′); Glucose unit δ_H 4.97 (1H, *d*, *J*=7.2 Hz, H-1′′), 3.20 (# H-2′′), 3.25 (# H-3′′), 3.15 (# H-4′′), 3.40 (# H-5′′), 3.48 (# H-6′′a), 3,67 (1H, *dl*, *J*=10.5 Hz, H-6′′b), 12.03 (O<u>H</u>-5). ^{1}H NMR (CD_3OD – $CDCl_3$ drops): Flavonoid unit δ_H 5.46 (1H, *dd*, *J*=13.1 and 3.2 Hz, H-2), 2.83 (1H, *dd*, *J*=17.3 and 3.2 Hz, H-3a), 3.11 (1H, *dd*, *J*=17.1 and 12.9 Hz, H-3b), 6.19 (1H, *d*, *J*=2.4 Hz, H-6), 6.21 (1H, *d*, *J*=2.4 Hz, H-8), 7.47 (1H, *m*, H-2′), 7.42 (1H, *m*, H-3′), 7.42 (1H, *m*, H-4′), 7.42 (1H, *m*, H-5′), 7.47 (1H, *m*, H-6′); Glucose unit δ_H 4.96 (1H, *d*, *J*=7.2 Hz, H-1′′), 3.35 (# H-2′′), 3.35 δ_H), 3.55 (# H-4′′), 3.35 (# H-5′′), 3.74 (1H, *dl*, *J*= 11.7 Hz, H-6′′a), 3,87 (1H, *dl*, *J*= 11.7 Hz, H-6′′b). ^{13}C NMR (DMSO-d6): Flavonoid unit δ_C 78.7 (C-2), 42,3 (C-3), 196,9 (C-4), 162,3 (C-5), 95,6 (C-6), 165,4 (C-7), 96,7 (C-8), 163,0 (C-9), 103,4 (C-10); Glucose unit δ_C 138,8 (C-1′), 126,8 (C-2′), 128,7 (C-3′), 128,8 (C-′4), 128,7 (C-5′), 126,8 (C-6′), 99,7 (C-1′′), 73,1 (C-2′′), 76,4 (C-3′′), 69,5 (C-4′′), 77,2 (C-5′′), 60,7 (C-6′′). COSY ^{1}H-^{1}H (DMSO-d6) most important correlations: Aromatic hydrogens H-2′ to -H-6′ on ring B; H-3a, H-3b and H-2 on ring C; H-6 and H-8 on ring B;

anomeric hydrogen H-1′′ and H-2′′ (glucose unit). HMBC (DMSO-d6) most important correlations: H-6 and C-5 (but not H-8 and C-5) on ring A; O<u>H</u>-5 and C-5 (but not O<u>H</u>-5 and C-9) on ring A; anomeric hydrogen H-1′′ (glucose unit) and C-7 (ring A) (# superimposed signals).

Pinocembrin-7-O-[4′′,6′′-HHDP]-β-glucose (**S-5**) ^{1}H NMR (DMSO-d6): Flavonoid unit δ_H 5.67 (1H, dd, *J*= 12.5 and 2.9 Hz, H-2), 2.85 (1H, dd, *J*= 16.8 and 3.0 Hz, H-3a), 3.35 (1H, *dd*, *J*=16.8 and 12.5 Hz, H-3b), 6.25 (1H, *d*, *J*=2.1 Hz, H-6), 6.20 (1H, *d*, *J*=2.1 Hz, H-8), 7.53 (1H, *m*, H-2′), 7.43 (1H, *m*, H-3′), 7.43 (1H, *m*, H-4′), 7.43 (1H, *m*, H-5′), 7.53 (1H, *m*, H-6′), 12.0 (*sl*, O<u>H</u>). Glucose unit δ_H 5.12 (1H, *d*, *J*=7.5 Hz, H-1′′), 3.35 (# H-2′′), 3.35 (# H-3′′), 4.60 (1H, *t*, *J*=9.8 Hz, H-4′′), 4.17 (1H, *dd*, *J*=9.8 and 6.4 Hz, H-5′′), 3.71 (1H, *dl*, *J*=13,5 Hz, H-6′′a), 4.95 (1H, *dd*, *J*=13.5 and 6.4 Hz, H-6′′b); HHDP unit δ_H 6.52 (H-3), 6.33 (H-3′). ^{13}C NMR (DMSO-d6): Flavonoid unit δ_C 78.8 (C-2), 42.3 (C-3), 197.0 (C-4), 162.8 (C-5), 95.7 (C-6), 165.4 (C-7), 96.7 (C-8), 163.1 (C-9), 103.6 (C-10), 138.6 (C-1′), 126.9 (C-2′), 128.8 (C-3′), 128.9 (C-4′), 128.8 (C-5′), 126.9 (C-6′); Glucose unit δ_C 99.7 (C-1′′), 73.8 (C-2′′), 73.8 (C-3′′), 71.6 (C-4′′), 71.1 (C-5′′), 62.9 (C-6′′); HHDP unit δ_C 115.7 (C-1), 124.5 (C-2), 106.3 (C-3), 144.4 (C-4), 135.4 (C-5), 144.7 (C-6), 167.2 C=O, 115.4 (C-1′), 124.8 (C-2′), 105.6 (C-3′), 144.3 (C-4′), 135.1 (C-5′), 144.6 (C-6′), 168.0 C=O′. COSY ^{1}H-^{1}H (DMSO-d6) most important correlations: Aromatic hydrogens H-2′ to H-6′ on ring B; H-3a, H-3b and H-2 on ring C; H-6 and H-8 on ring B; anomeric hydrogen H-1′′ and H-2′′ on glucose unit. HMBC (DMSO-d6) most important correlations: Anomeric hydrogen H-1′′ and C-7 (ring A), HHDP CO′ and HHDP H-3′, HHDP CO′ and HHDP H-6a′′ (glucose unit) and H-6b′′ (glucose unit), HHDP CO and HHDP H-3, HHDP CO and HHDP H-4′′ (glucose unit) (# superimposed signals).

Ursolic acid ^{1}H NMR ($CDCl_3$): δ_H 5.50 (1H, t, *J*=3.6 Hz, H-12); ^{13}C NMR ($CDCl_3$): δ_C 39.3 (C-1), 28.2 (C-2), 78.3 (C-3), 39.5 (C-4), 55.9 (C-5), 18.9 (C-6), 33.7 (C-7), 40.1 (C-8), 48.2 (C-9), 37.4 (C-10), 23.8 (C-11), 125.8 (C-12), 139.4 (C-13), 42.6 (C-14), 28.8 (C-15), 25.1 (C-16), 48.2 (C-17), 53.7 (C-18), 39.5 (C-19), 39.6 (C-20), 31.2 (C-21), 37.6 (C-22), 29.0 (C-23), 16.7 (C-24), 15.8 (C-25), 17.6 (C-26), 24.1 (C-27), 180.1 (C-28), 17.6 (C-29), 21.6 (C-30).

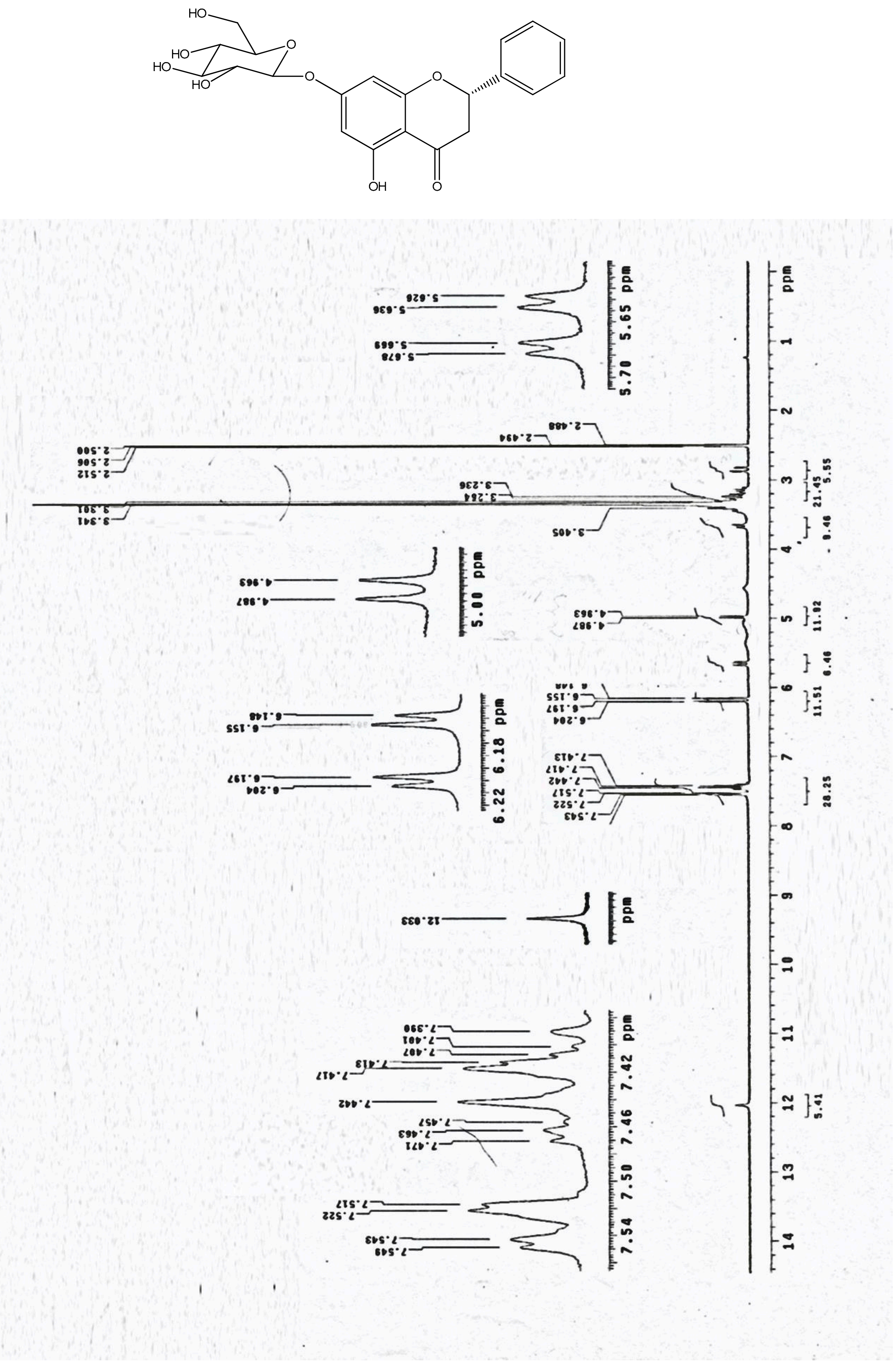

Figure 1. ^{1}H NMR spectrum of **S4** (δ_H, DMSO-d_6, 75 MHz).

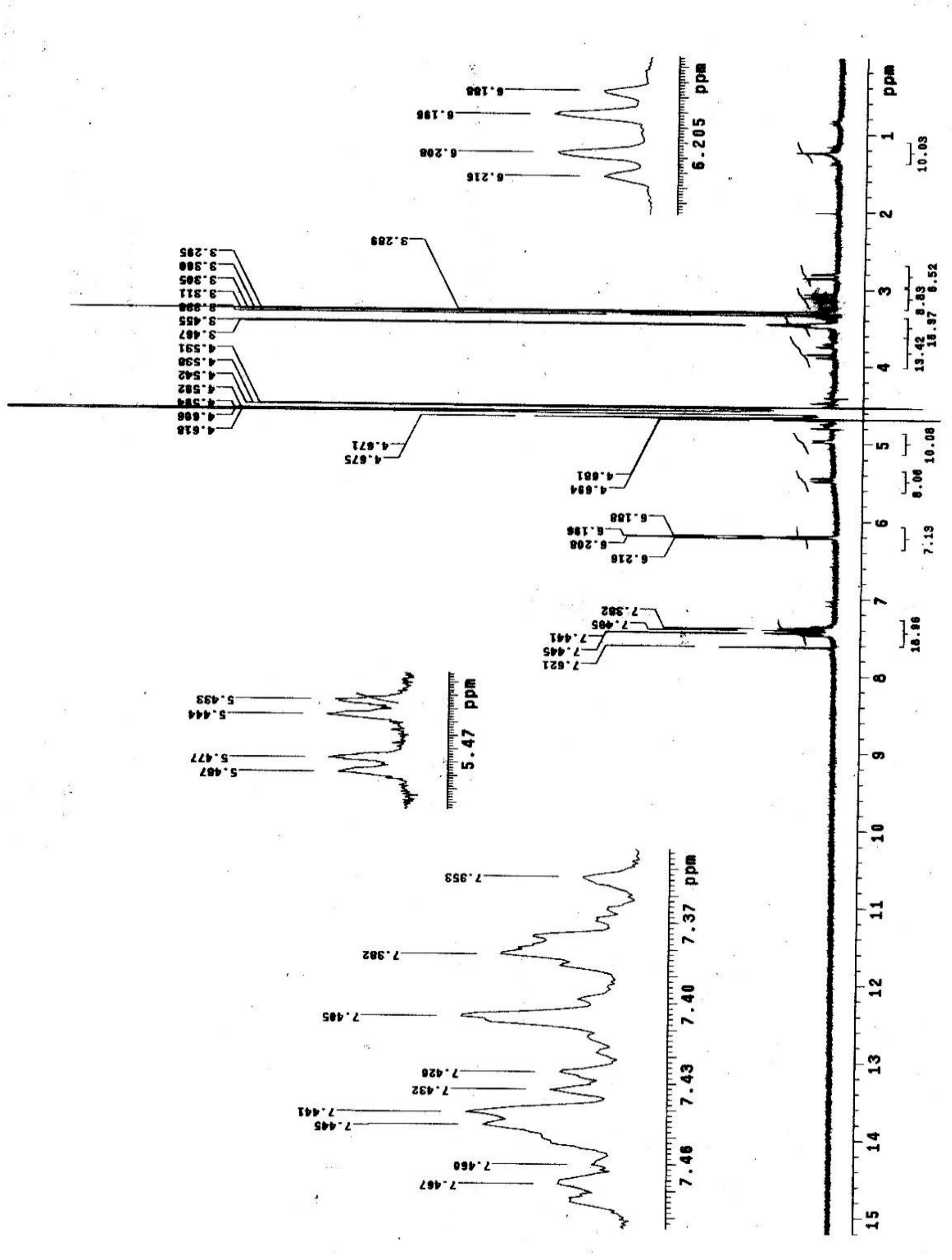

Figure S2. ^{1}H NMR spectrum of **S4** (δ_H, CD_3OH-$CDCl_3$ drops, 75 MHz).

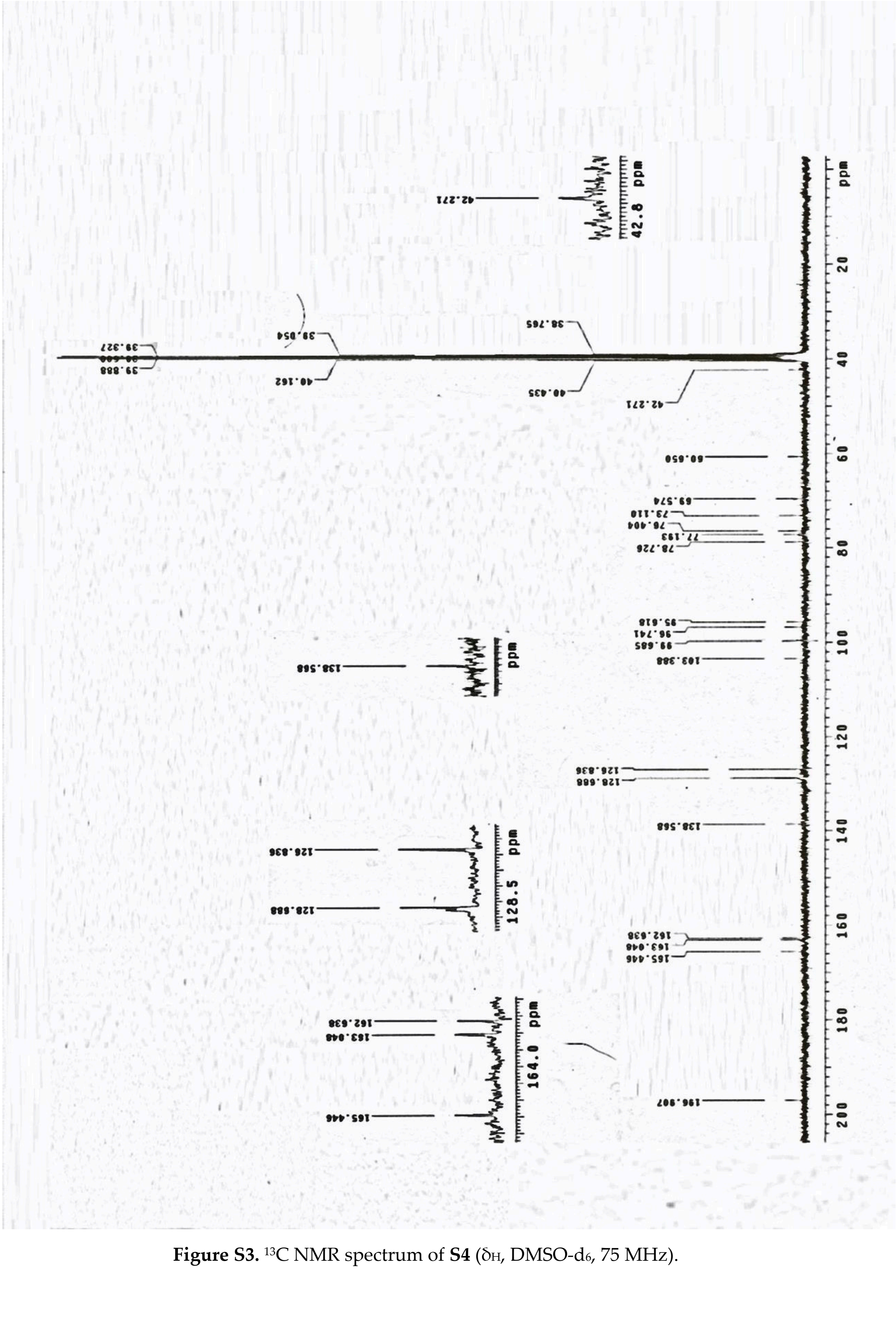

Figure S3. ^{13}C NMR spectrum of **S4** (δ_H, DMSO-d_6, 75 MHz).

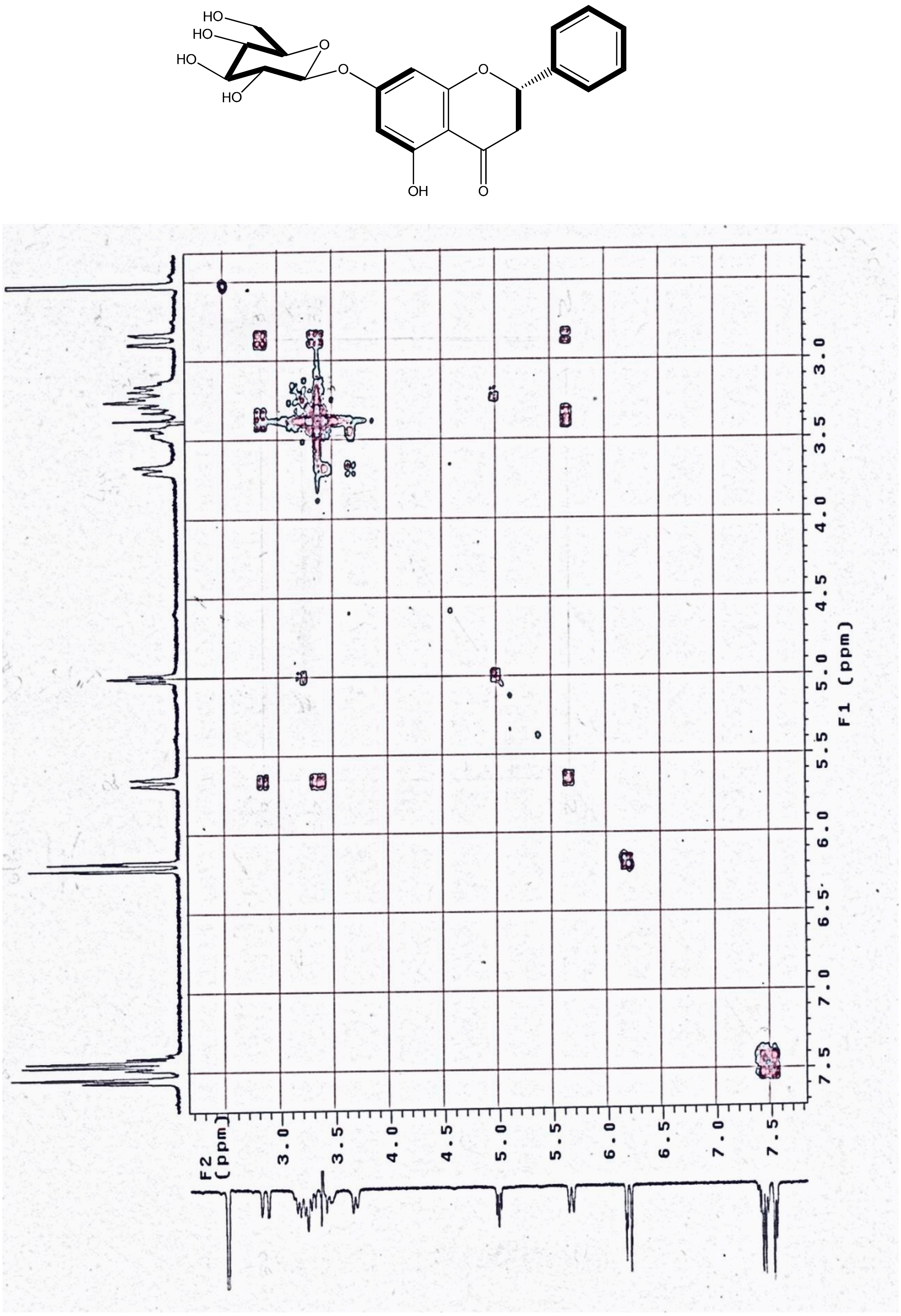

Figure S4. COSY H-H of **S4** (δ_H, DMSO-d_6) and most important correlations.

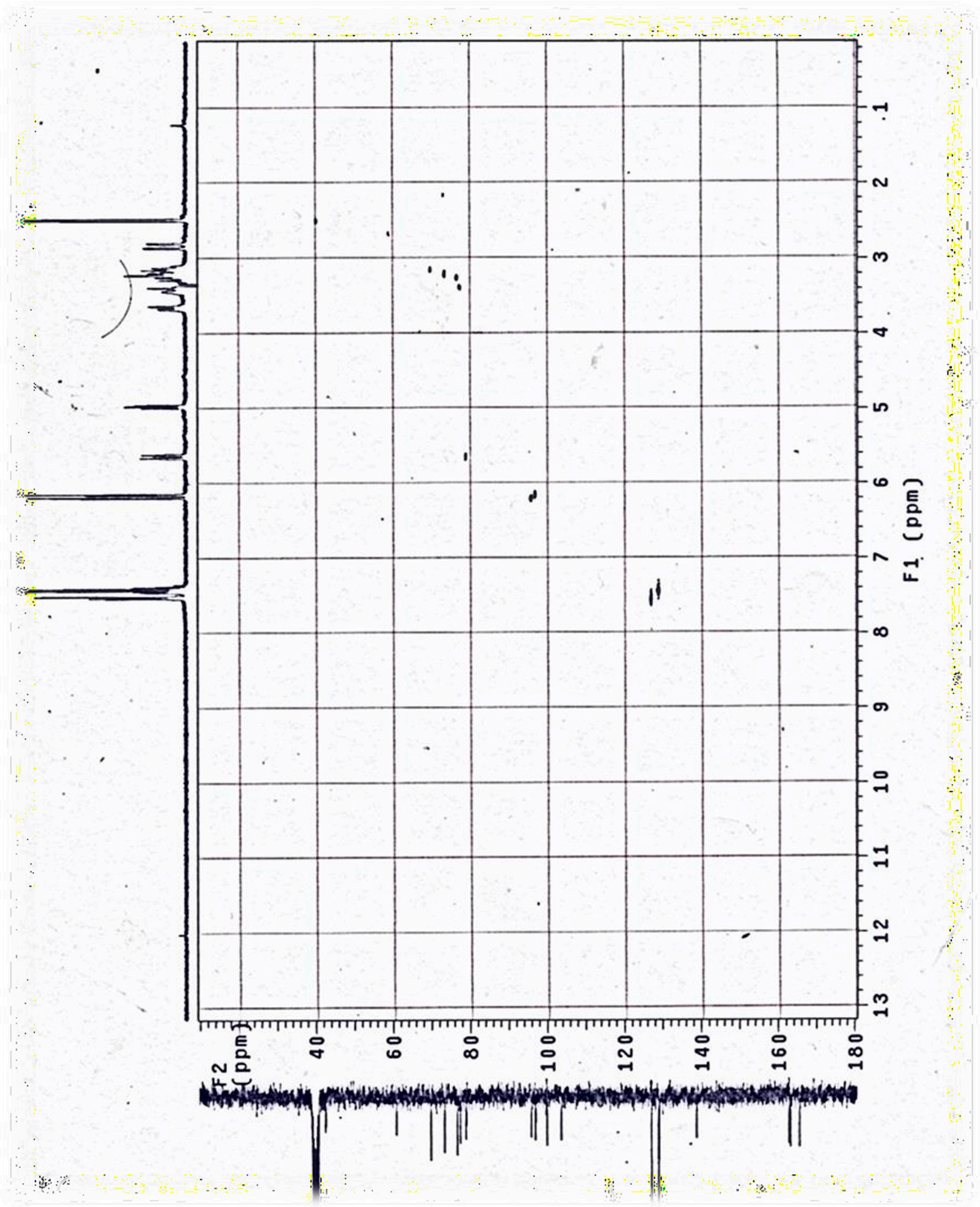

Figure S5. HETCO of **S4** (δ_H, DMSO-d_6).

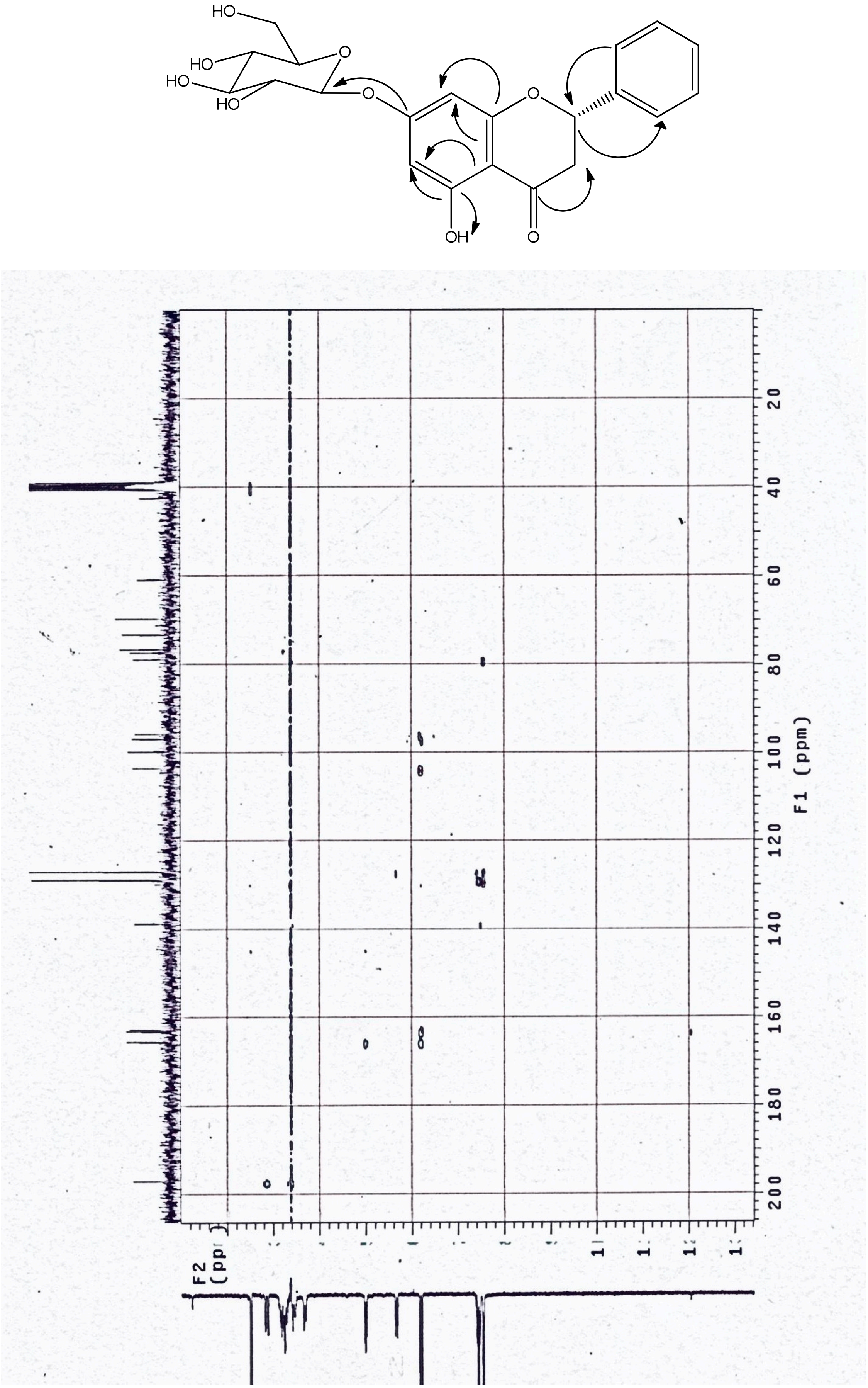

Figure S6. HMBC of **S4** (δ_H, DMSO-d_6) and most important correlations.

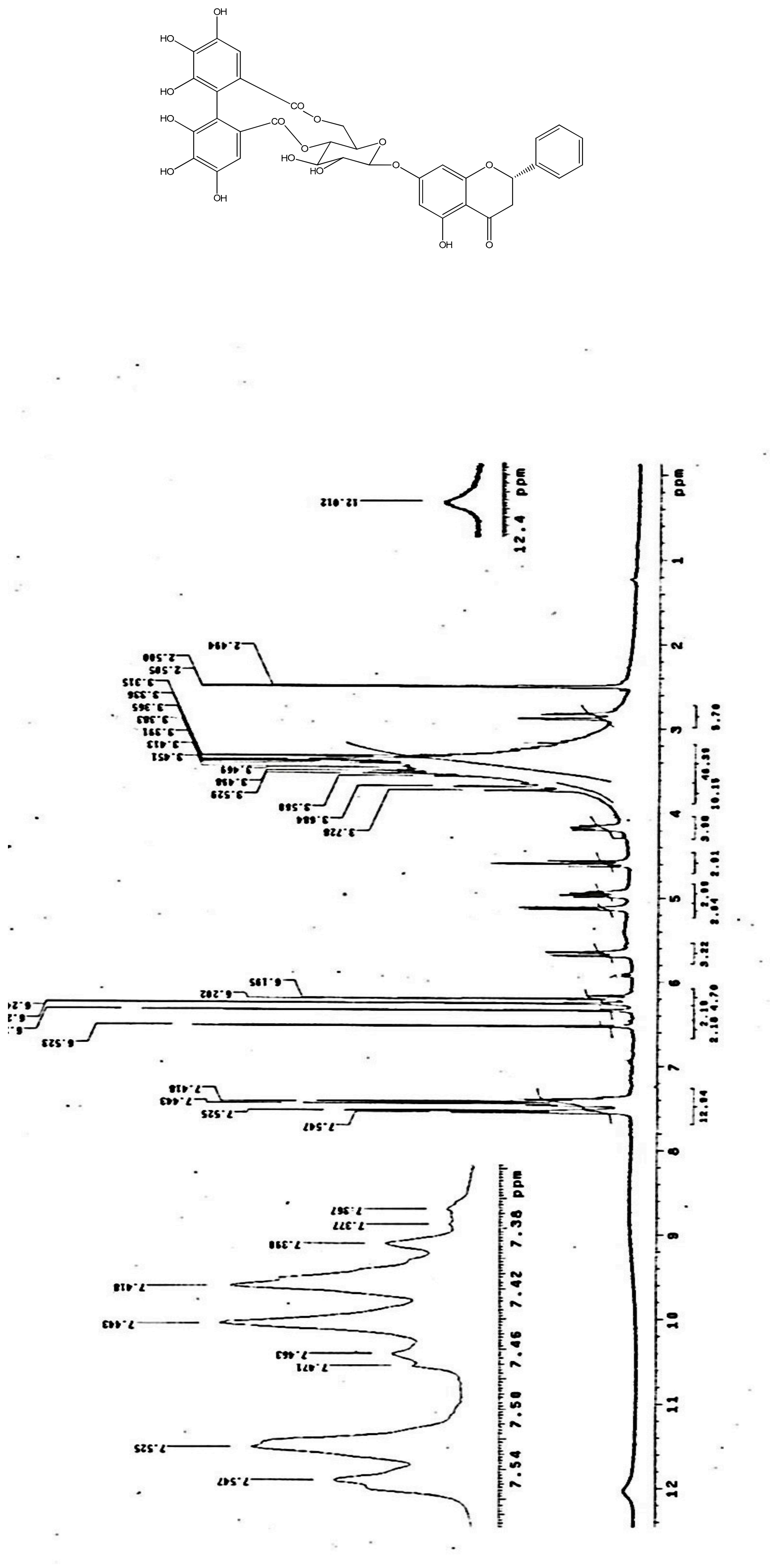

Figure S7. ^{1}H NMR spectrum of **S5** (δH, DMSO-d$_6$, 75 MHz).

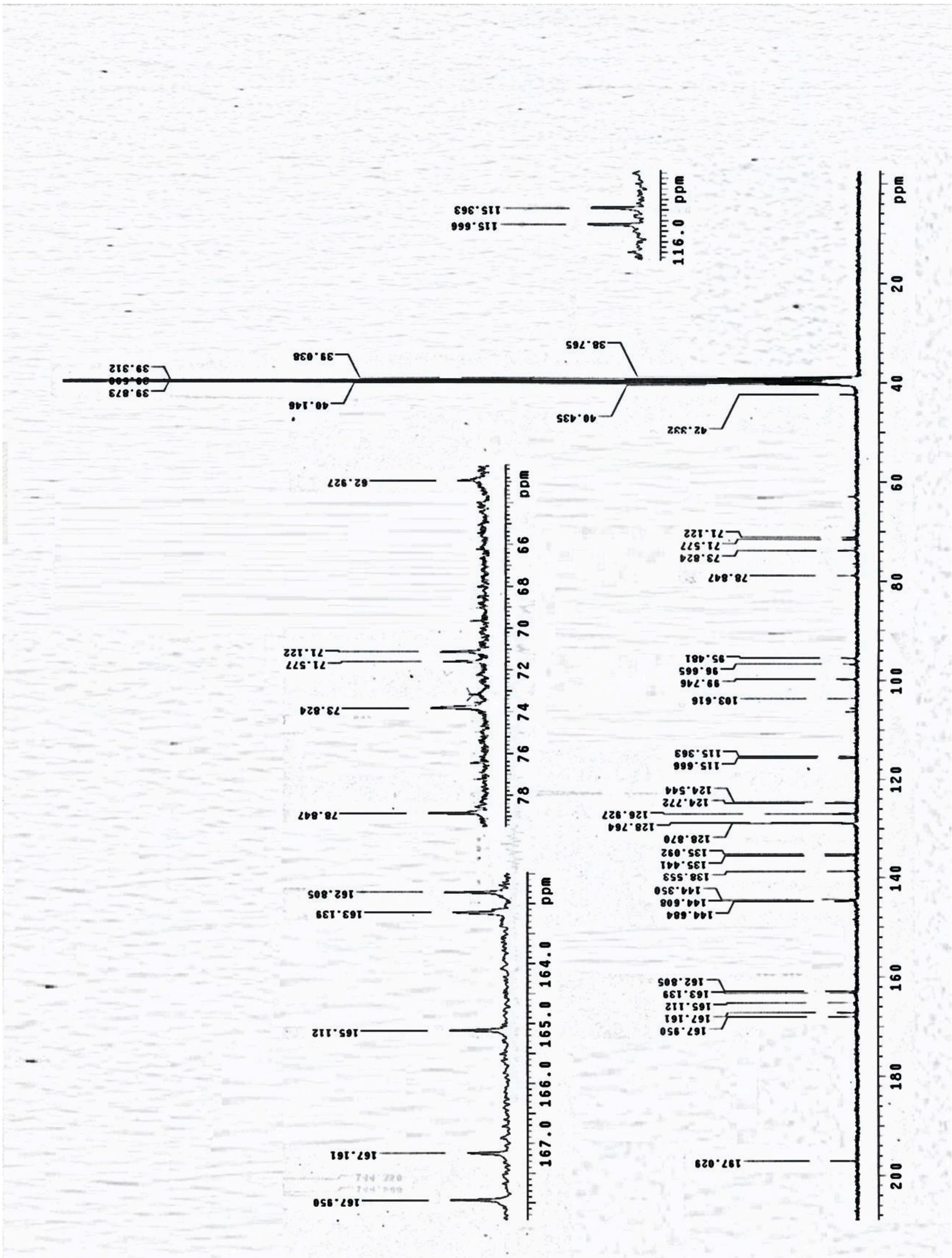

Figure S8. ^{13}C NMR spectrum of **S5** (δ_C, DMSO-d_6, 75 MHz).

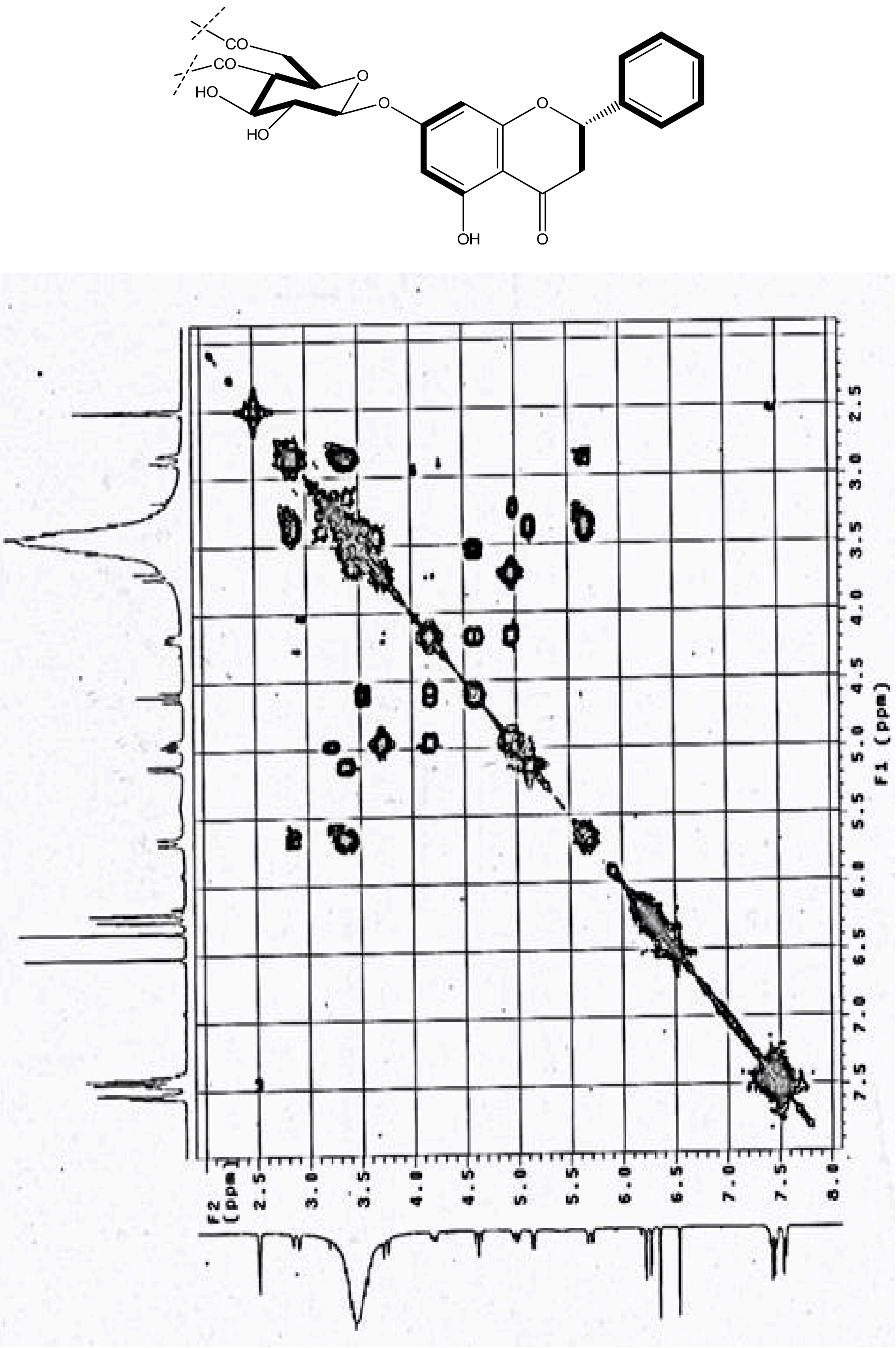

Figure S9. COSY H-H of **S5** (δ_H, DMSO-d_6) and most important correlations.

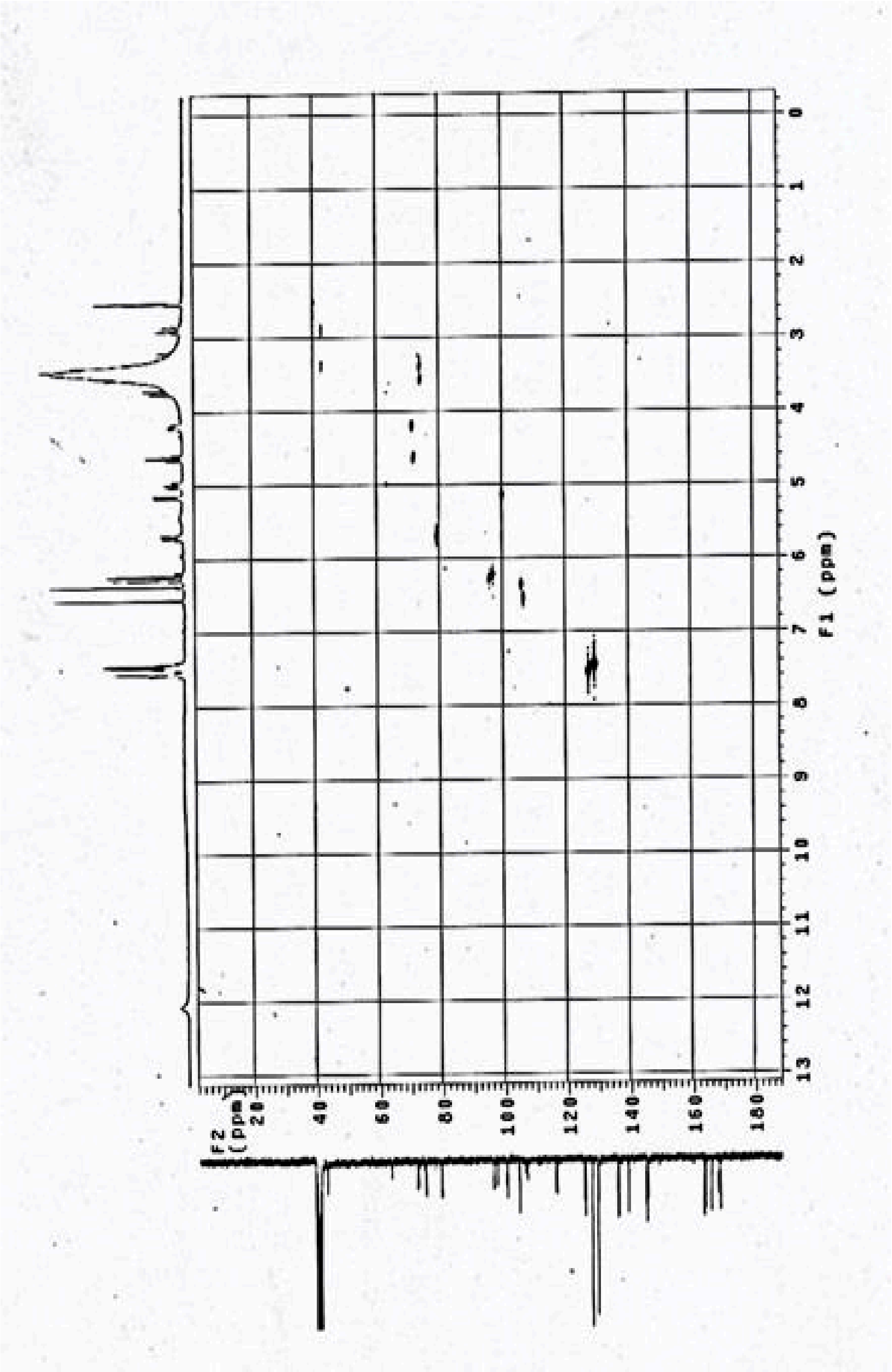

Figure S10. HETCO of **S5** (δ_H, DMSO-d_6).

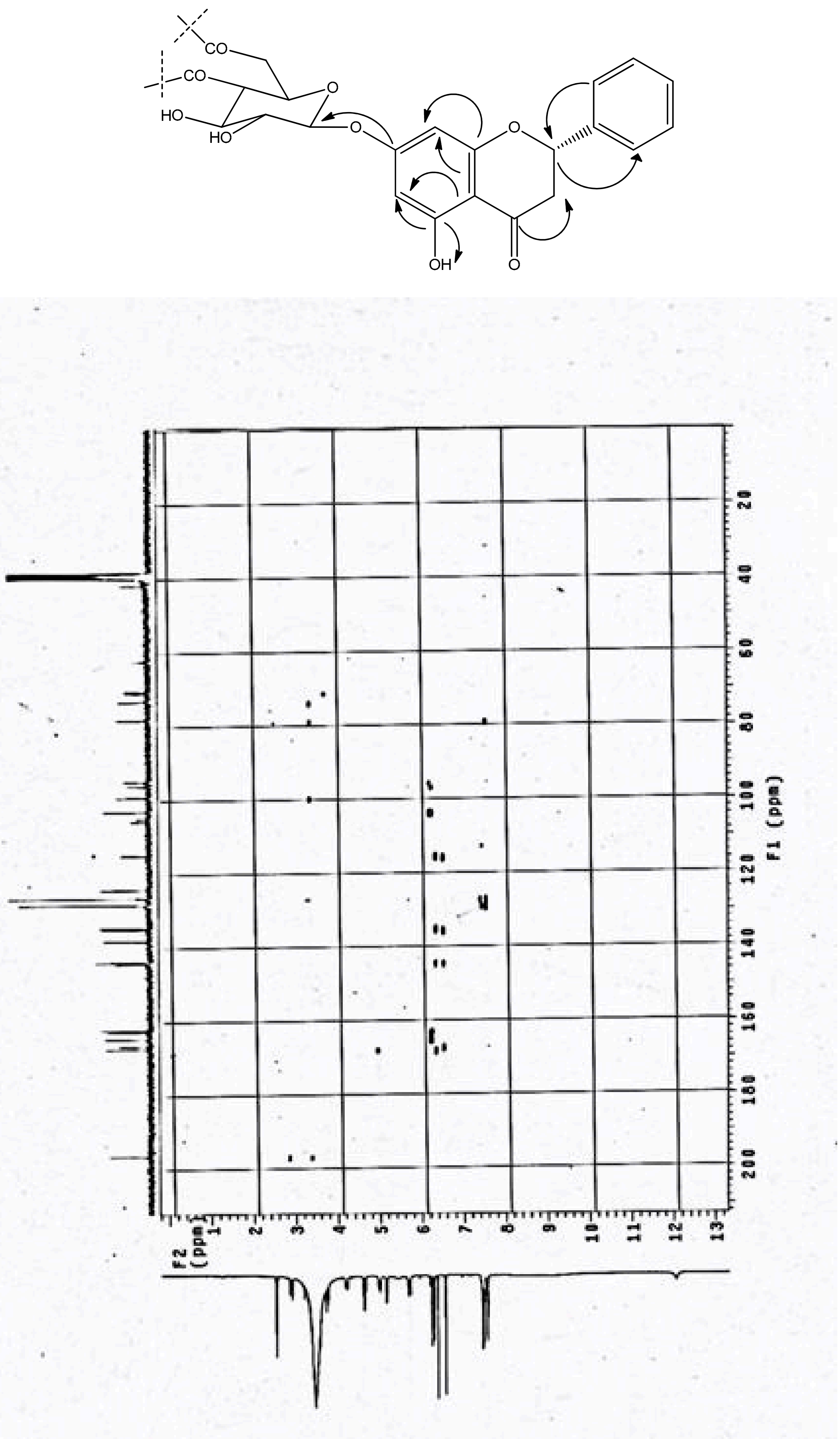

Figure S11. HMBC of **S5** (δ_H, DMSO-d_6).
